# Roles of oxides of nitrogen on quality enhancement of soybean sprout during hydroponic production using plasma discharged water recycling technology

**DOI:** 10.1038/s41598-018-35385-5

**Published:** 2018-11-15

**Authors:** Eun-Jung Lee, Muhammad Saiful Islam Khan, Jaewon Shim, Yun-Ji Kim

**Affiliations:** 1 Faculty of Food Science and Biotechnology, College of Life Science, Sejong University, Seoul, 05006 Republic of Korea; 2 Division of Food Safety and Distribution, Korea Food Research Institute, Wanju-Gun, Jeollabuk-Do 55365 Republic of Korea; 3 Department of Food Biotechnology, University of Science and Technology, Daejeon, 305-350 Republic of Korea

## Abstract

This study was performed to assess the effect of plasma-discharged water recycling technology as irrigation water on soybean sprout production. Two different types of irrigation water were used individually for cultivation, including plasma discharged water as a source of oxides of nitrogen and tap water, irrigation water was recycled for every 30 minutes. Plasma discharged irrigation water reduced overall 4.3 log CFU/ml aerobic microbe and 7.0 log CFU/ml of artificially inoculated *S*. Typhimurium within 5 minutes and 2 minutes, respectively, therefore sprout production occurs in a hygienic environment. Using of plasma-discharged water for cultivation, increases the amount of ascorbate, asparagine, and γ-aminobutyric acid (GABA) significantly (*p* < 0.05), in the part of cotyledon and hypocotyl of soybean sprout during 1 to 4 days of farming. A NO scavenger, 2-(4-carboxy-phenyl)-4,4,5,5-tetramethylimidazoline-1-oxy-3-oxide (cPTIO), was added in irrigation water to elucidate the roles of the oxides of nitrogen such as NO_3_^−^, NO_2_^−^ generated in plasma discharged water. It was observed that all three nutrients decreased in the cotyledon part, whereas ascorbate and GABA contents increased in the hypocotyl and radicle part of bean sprout for the same duration of farming. The addition of NO scavenger in the irrigation water also reduced growth and overall yield of the soybean sprouts. A recycling water system with plasma-discharged water helped to reduce the amount of water consumption and allowed soybean sprouts growth in a hygienic environment during the hydroponic production.

## Introduction

Soybean sprouts are a nutritious vegetable that have been widely consumed in China, Japan and Korea since B.C. 1–935 and the beginning of Goryeo era (918–1392)^1^. The hydroponic production of soybean sprouts is popular for enhancing their nutritional value, and it allows for cultivation throughout the year^2^. The germination of soybeans increases the amount of some nutrients, such as vitamins and asparagine^3,4^, whereas cultivation in warm and humid environments may increase the microbial amount, which may lead to lessen the product yield and quality^5–7^. Alterations of cultivation conditions can affect plant growth and the biosynthesis of nutrients, for instance ascorbate and γ-aminobutyric acid (GABA)^8–11^. Additionally, hydroponic sprout production requires a large amount of water from the cultivation stage to the packaging stage. Therefore, environmentally friendly and economic sprouting technologies are necessary to enhance the nutritional quality, improve the growth and yield of soybean sprouts. Application of plasma discharged water recycling technology can overcome all the constraints stated above. Recycling water system can reduce the water consumption that makes the production system economic and introducing of disinfection technology can reduce the spread of unwanted viruses and food-borne pathogens in the plant^12^. Furthermore, presence of different radicals in the irrigation water increases the nutritional quality of bean sprout specially oxides of nitrogen.

A wide variety of plasma technologies has been studied in the context of microbial reduction in food^13,14^, environment^15,16^ and wound healing^17^. Reactive oxygen species (ROS), reactive nitrogen species (RNS) and their reaction products such as ozone, hydrogen peroxide, nitrous acid and nitric acid generates by plasma^18,19^, which are soluble in water and are capable of biological sterilization^20^. Our previous research showed the effectiveness of different types of plasma, including arc and dielectric barrier discharge (DBD), on viral and pathogenic inactivation in refs^14–16,20–22^. Plasma discharged water has the ability to reduce microbial contamination from the different types of food surfaces including onion, perilla leaf, lettuce etc., without changing the physiochemical properties of the treated sample^14,21^.

Plasma technology has also been introduced in botanical research to stimulate seed germination processes and plant growth. For instance, air plasma irradiation is used on *Arabidopsis thaliana* (L.) seeds^23^ and *Pharsalus vulgaris* beans^24^. In addition, the He plasma irradiation of wheat^25^ and soybean^26^ seeds is commonly used. These studies indicate that implementation of plasma technology raises the hydrophilicity of the seed surface^24,27^ and hence, the stimulation of seed germination occurs. Sarinont *et al*. (2016) reported that plasma irradiation more effectively enhances plant growth in humid air than in dry air^28^. Park *et al*. (2013) showed the potential implementation of plasma in water treatment for plant growth and showed various effects of plasma-discharged water on different plants^29^. Use of the hydroponic method for soybean sprout cultivation water supply is prime aspects that have direct impact on safety, growth and nutritional content. Vitamin C, important ingredient present in fresh produces, mainly as ascorbic acid and ascorbate, is a common component that is very popular due to its free radicals scavenging capability^30^. Plasma-discharged water mainly contains various free radicals, such as ROS and RNS; hence, it is very important to analyze the content of ascorbate in beansprouts cultivated with plasma-discharged water. Asparagine is another important nutrient in soybean sprouts due to its acetaldehyde detoxifying ability^6^. GABA has many functions in human health, such as certain forms of cancer preventing ability and dropping the danger of cardiac ailments by regulating blood pressure and heart beats^31,32^. Accumulations of GABA content in plant materials helps to control plant growth in reply to biotic and abiotic strain^33,34^. Soybean sprouts are rich in GABA content, which can vary depending on the irrigation conditions^35^. Hence it is very important to monitor the effect of plasma discharged water on the changes of asparagine and GABA contents too.

There are various effects of plasma-treated water on plant growth and nutritional content due to the presence of different radicals in plasma-treated water^36^. H_2_O_2_ and NOx act as molecular signals, helps to break seed dormancy and synthesizing cellular antioxidants^37^. Exogenous NO generated with sodium nitroprusside helps to induce seed germination or to reduce dormancy in the presence of various abiotic stresses^38^. The exogenous application of H_2_O_2_ on pea seedlings also plays an important role to control seed dormancy time by decreasing abscisic acid^39^. Different environmental stimuli have been used to raise amount of phytochemical in the plant materials^40^. Therefore, the use of plasma-discharged water recycling technology could be vital for soybean sprout cultivation in the context of its quality enhancement, growth, yield, and stimulation of seed germination processes. However, the roles of plasma-discharged water on seed sprouting, plant growth, yield and nutritional value have not yet been studied. So, it is imperative to know the roles of individual radicals, especially soluble nitrite/nitrate anions on plant growth and nutritional variation. The radical scavenging method is a passive technique that may help us to understand how specific radicals in plasma-treated water affect plant growth and nutritional content variation. In this study, hydroponic soybean sprout was cultivated for the first time to investigate the roles of soluble nitrite/nitrate anions on nutrient content, such as changes in ascorbate, asparagine, and GABA, by varying the irrigation water and adding NO scavenger during soybean sprout cultivation.

## Results

### Content of radicals in plasma-discharged water and their roles

**Figure 1:**
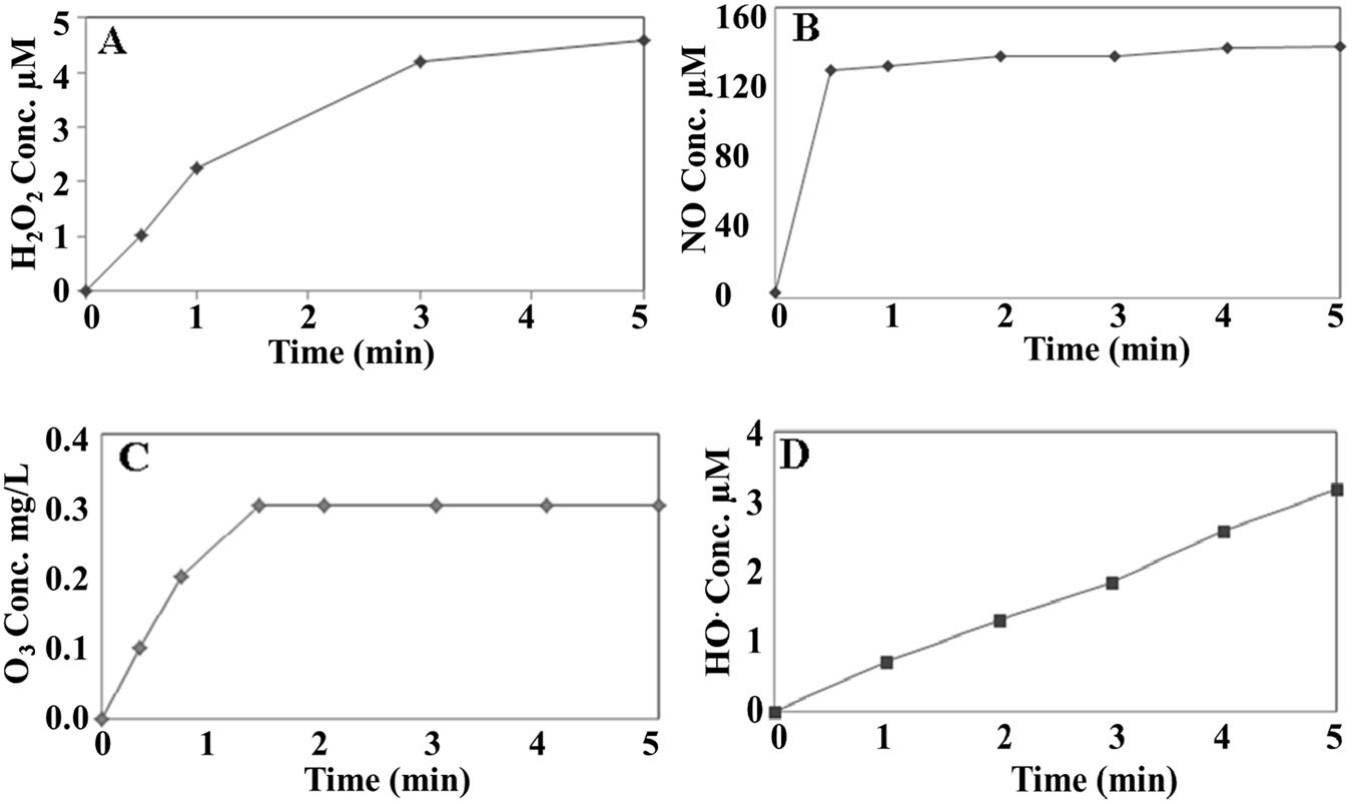
Dissolved ozone (**A**), OH radical (**B**), H_2_O_2_ (**C**), and NO (**D**) concentration produced by the DBD plasma.

DBD plasma generates reactive oxygen species (ROS) and reactive oxygen species (RNS) that confirmed from the optical emission spectra figure (figure was published elsewhere)^20^. The generated species were dissolved in water as the plasma jet was passed through water. In aqueous medium, nitric oxide is a highly reactive species that may react with oxygen to produce nitrite and stays as an unstable nitrous acid (HNO_2_). HNO_2_ decompose rapidly into nitrogen dioxide and reacts with hydroxyl radicals to produce peroxynitrous acid which is not stable in acidic conditions and changes into stable nitrate (NO_3_^−^)^41^. Hence plasma discharged water contains nitrite/nitrate anions (NO_2_/NO_3_). However, throughout the manuscript NOx represents nitrite/nitrate anions if not mentioned otherwise. Figure 1 shows the H_2_O_2_, NOx, dissolved O_3_ and OH radical concentrations in the plasma-discharged water at different plasma treatment times. The O_3_ concentration reached its highest level within 2 minutes of plasma treatment. The OH radical and H_2_O_2_ concentrations increased with increasing treatment time, up to 5 minutes, and the NOx concentration peaked after 1 minute. After 5 minutes of plasma operation, the water contained 0.3 ppm of O_3_, 3.2 µM of OH radical, 4.6 µM of H_2_O_2_ and 150 µM of oxides of nitrogen (NOx), and the value remained constant until the plasma device remained switch on (except OH radical). The detail phenomenon for OH radical concentration was described in our previous study^20^.

### Microbial inactivation ability of irrigation water

**Figure 2:**
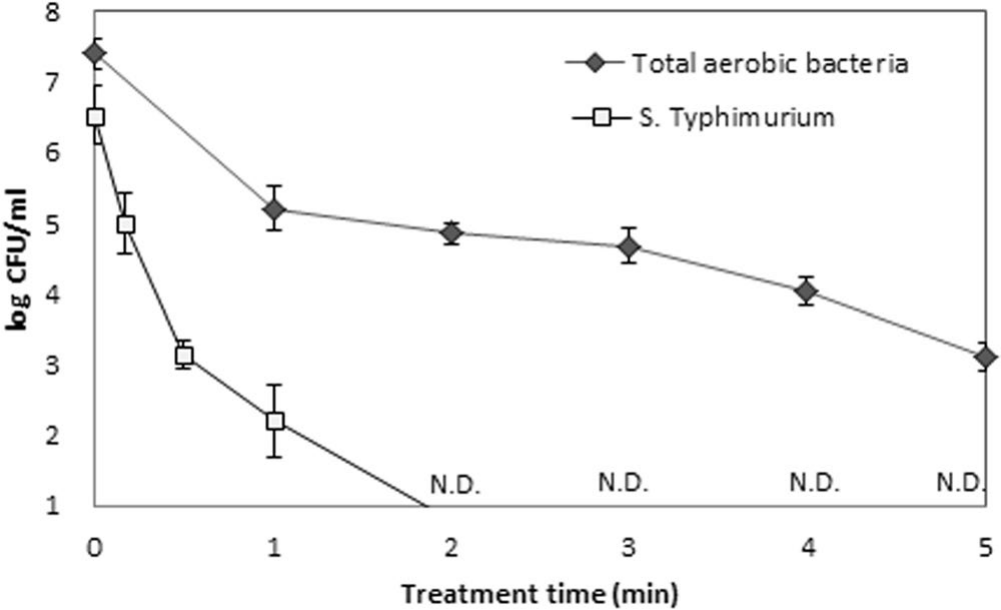
The reduction effect of DBD plasma for the total aerobic bacteria and the inoculated *S*. Typhimurium ATCC14028 in irrigation water (after 24 hours of recycling) was evaluated.

The hydroponic production of soybean sprouts provides very favorable conditions for microorganism growth, especially when the irrigation water is recycled. Hence, the microbial reduction potential of the DBD plasma generator for a recycled irrigation water system was assessed. After 24 hours of cultivation, the total aerobic bacterial count increased by approximately 7.0 log CFU/ml in the irrigation water. Plasma treatment was performed on 24 hours recycled water, overall 4.3 log CFU/ml aerobic microbial reduction was obtained within 5 minutes, while 7.0 log CFU/ml of artificially inoculated *S*. Typhimurium inactivation was obtained within 2 minutes Fig. 2.

### The growth percentage and yield of soybean sprouts

**Figure 3:**
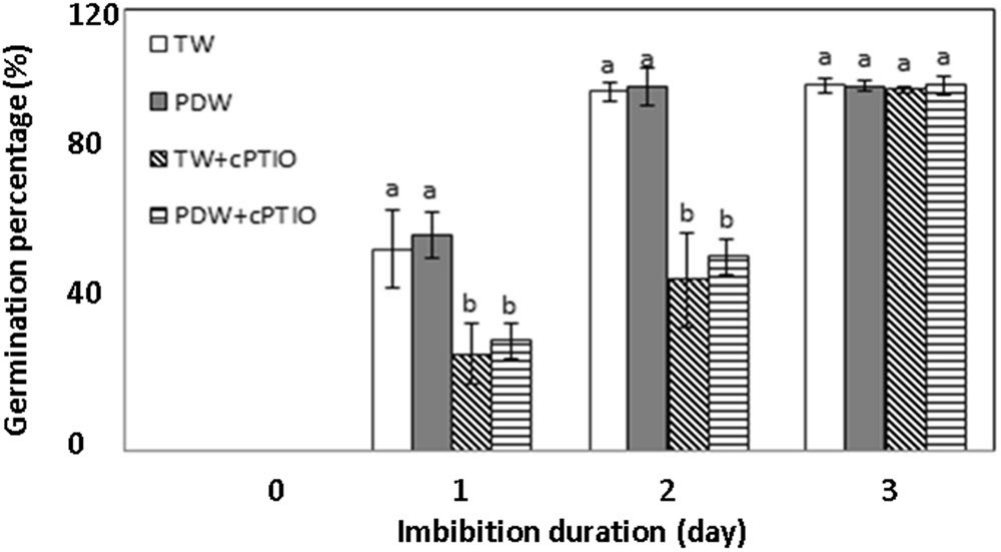
The changes in the germination percentages during soybean sprout cultivation depending on the irrigation water. TW and PDW represent tap water and plasma-discharged water, respectively. The addition of cPTIO is indicated by +cPTIO in TW or PDW. Treatments with different letters (a, b) for the same imbibition time were significantly different based on Duncan’s multiple range test (*p* < 0.05).

To understand the effect of irrigation water on soybean sprout growth and yield, two different types of irrigation water, plasma-discharged water and tap water, were used in the recycled water system for the hydroponic production. The germination percentage of soybean seeds and the individual weight of each part (cotyledon, hypocotyls, and radicle) of the harvested soybean sprouts were measured at different stages of sprout production to assess the effect of each type of irrigation water. Figure 3 shows the change in germination percentage depending on cultivation water. The germination percentage of soybean seeds was investigated after imbibition for 24, 48 and 72 hours. The germination percentage reached almost 99% within 48 h of imbibition regardless of the irrigation water used, and no substantial variation was detected in the rate of germination until 72 hours of imbibition. Figure 3 shows that addition of an NO scavenger blocks the germination process during the early stages of soybean imbibition. After 24 hours of imbibition, 26–30% radicle emergence was observed in presence of cPTIO, which is less than the germination observed in absence of cPTIO in the irrigation water. The observed germination percentages of soybeans after 72 hours of imbibition were almost 99% with or without cPTIO regardless of the irrigation water used in this study.

**Figure 4:**
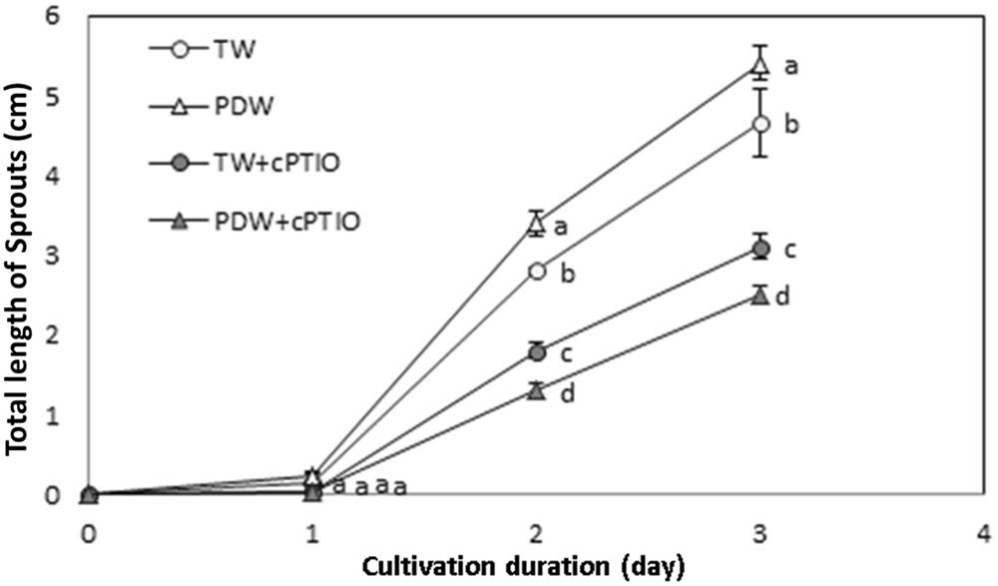
The changes in total length during soybean sprout cultivation depending on the cultivation water. Treatments with different letters (a, b, c, d) for the same cultivation day were significantly different based on Duncan’s multiple range test (*p* < 0.05).

The result of irrigation water on the length of individual soybean sprout parts was measured after 72 hours of cultivation, because distinguishing hypocotyls and radicles at the early stages of growth is difficult (Fig. 4). At days 2 and 3, the total length of the soybean sprouts cultivated with plasma-discharged water was significantly larger as compare to the sprout cultivated with tap water (*p* < 0.05) (Fig. 1SA and B). The growth of soybean sprouts reduced in the presence of cPTIO for both types of irrigation water used in this water recycling system during hydroponic soybean sprout production (*p* < 0.05).

The effects of the plasma-discharged water on the total weight and the total length of two different parts of the soybean sprouts were evaluated after 72 hours of cultivation (Table 1). The total weight and the length both increased significantly in the soybean sprouts cultivated with plasma-discharged water compared to the sprouts cultivated with tap water (*p* < 0.05). The presence of cPTIO in the cultivating water in both plasma-discharged and tap water showed a decreasing trend in the weight and length of the sprout. The observed differences in the radicle length in the presence of cPTIO for both types of cultivating water were not significant (p > 0.05) (Table 1).

**Table 1:** Changes in the average sprout weight and length after 4 days of soybean sprout cultivation depending on the type of irrigation water.

Cultivating water	Tap water	Plasma-discharged water	Tap water + cPTIO	Plasma-discharged water + cPTIO
Parameters				
**(Units: g)**				
Weight (g)	48.2 ± 1.1^b^	53.5 ± 1.5^a^	32.8 ± 0.8^c^	30.3 ± 1.0^c^
Hypocotyl length (cm)	5.2 ± 0.3^b^	6.3 ± 0.5^a^	3.1 ± 0.3^c^	2.6 ± 0.6^d^
Radicle length (cm)	5.5 ± 0.6^a^	5.8 ± 0.9^a^	4.3 ± 0.4^b^	4.0 ± 0.8^b^

Average and standard deviations with different letters (a, b, c, d) in the same line were significantly different based on Duncan’s multiple range tests (*p* < 0.05).

### Evaluation of nutrient content

#### Ascorbate content

**Figure 5:**
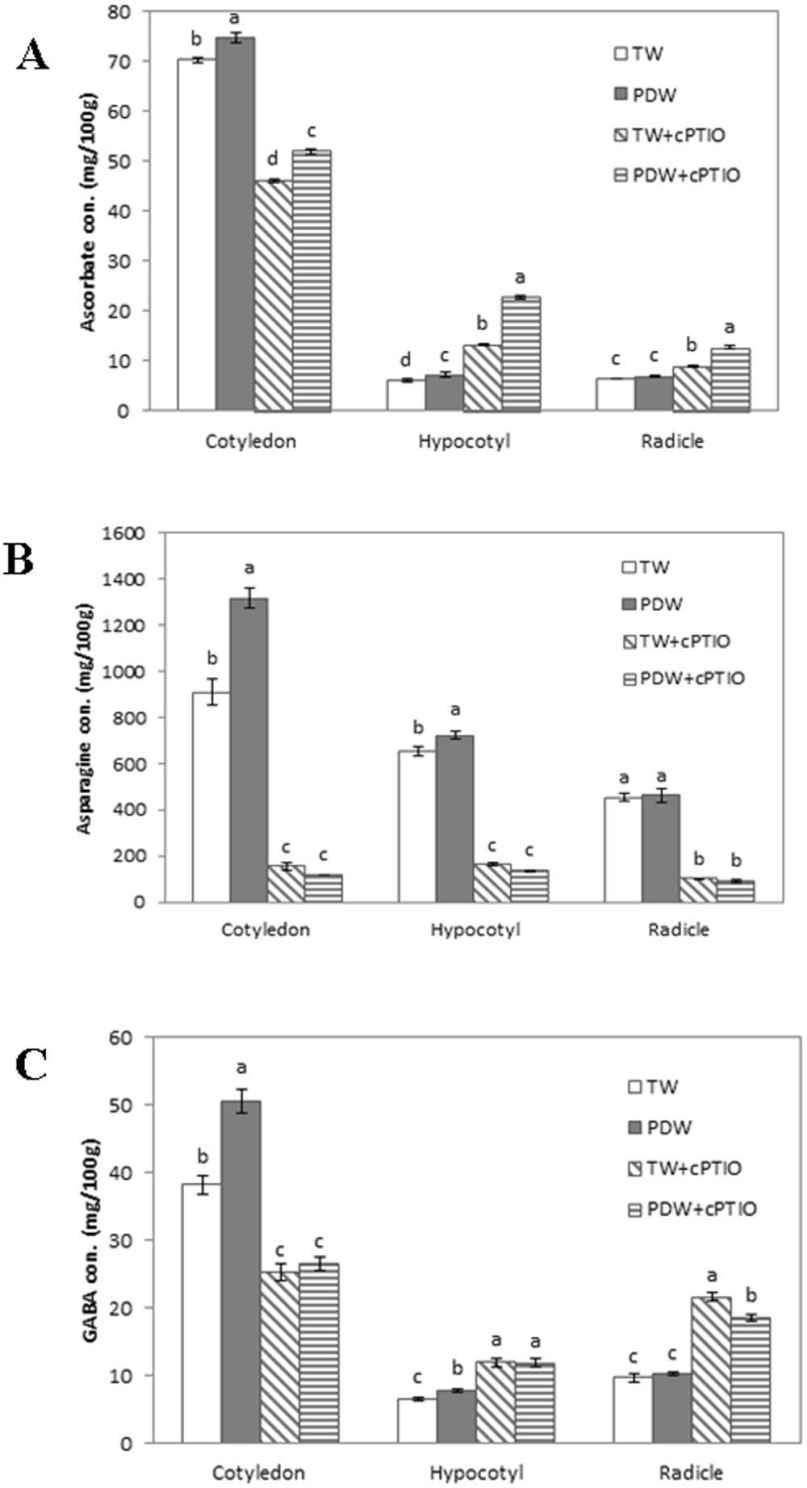
The changes in the ascorbate (**A**), asparagine (**B**), and GABA (**C**) contents in each plant part (100 g fresh weight) of the soybean sprouts depending on the type of irrigation water. Treatments with different letters (a, b, c, d) for the same plant part of the soybean sprout were significantly different based on Duncan’s multiple range test (*p* < 0.05).

Figure 5A shows the ascorbate content per 100 g of each part of the soybean sprout after 4 days of cultivation. The ascorbate content in the cotyledons and hypocotyls was significantly increased when the cultivation was performed with plasma-discharged water instead of tap water (*p* < 0.05). Variation in the cultivation water did not play any role in the ascorbate content of the radicle. However, in the hypocotyls and in the radicle, the ascorbate content increased in the presence of cPTIO.

#### Asparagine content

Figure 5B shows the asparagine content per 100 g of each part of the soybean sprout after 4 days of cultivation. The asparagine content in the cotyledons and hypocotyls significantly increased when the cultivation was performed with plasma-discharged water instead of tap water (*p* < 0.05), and the adding of cPTIO notably reduced the amount of asparagine. Irrigation water does not influence the amount of asparagine in radical but using of cPTIO in the irrigation water notable reduced the amount of asparagine in radical.

#### GABA content

Figure 5C shows the GABA content per 100 g of each part of the soybean sprout after 4 days of cultivation. The GABA content in the cotyledons and hypocotyls was significantly increased when the cultivation was performed with plasma-discharged water instead of tap water (*p* < 0.05). For the radicles, the amount of GABA content did not show any significant difference between the different types of cultivating water. The addition of cPTIO notably decreased the GABA content in the cotyledons but significantly increased the content in the hypocotyls and radicles with the addition of NO scavenger.

## Discussion

The antimicrobial capability of plasma discharged water is well-studied. Different species present in the plasma discharged water plays different role on microbial inactivation via different mechanism. Among the radicals present in plasma discharged water OH radicals have the highest inactivation ability with dissolved ozone placed in the second in ranking. The inactivation efficiency of H_2_O_2_ generated by our device was evaluated and no inactivation was obtained^20^. Because the amount of H_2_O_2_ produced by our device is lesser than the minimum amount of H_2_O_2_ necessary for the microbial decontamination by H_2_O_2_ alone. Although the antimicrobial capability of H_2_O_2_ is commonly used and well-studied^18^. Nevertheless, this study may not conclude the mutual sterilization effect of H_2_O_2_ with the other generated species. Generated oxides of nitrogen plays some effects on microbial inactivation either alone or with the combination of other radicals. The microbe’s experiences a strong effect with the radicals present in the plasma discharged water and become inactive through some chemical or physical process. Initially surface abrasion occurs which cannot not be repaired by living cells rapidly and hence inward flows of radicals occur to inactivate microorganism completely^42,43^. A significant amount of microbial inactivation obtained for the both background microorganism and pathogen as well (Fig. 2). The reduction of inoculated *S*. Typhimurium by DBD plasma was rapid compared to the total aerobic bacteria in the irrigation water. Plasma-discharged water flows continued in 30 minutes intervals for 30 seconds. During this process, the water recycling plasma device became activated in every 12 hours, and after 5 minutes it automatically became deactivated. Therefore, there was no possibility of microorganism growth/survival in the irrigation water during the hydroponic production of bean sprouts using plasma-discharged water recycling technology. The antimicrobial properties of plasma-discharged water show an improved performance in the context of achieving a healthy production environment for soybean sprout cultivation. Therefore, it can be stated that the plasma discharged water used in this study as irrigation water helps the sprouts to be grown in a hygienic environment. This result shows that using of plasma-discharged water could be beneficial for soaking soybean seeds during the germination process as well.

In this work, plasma device was activated for 5 minutes in every 12 hours, hence the possibilities of the presence of H_2_O_2_ and different oxides of nitrogen in the irrigation water throughout the cultivation is high as compare to the other radical generated. Generated O_3_ gas moved away from the water within couple of minutes (data not shown here) and the OH radical converts into H_2_O_2_ due to its short life time. Therefore, it is important to know the effects of H_2_O_2_ and NOx on sprout growth and its nutritional content. NOx generated in plasma-discharged water is an extremely reactive species. The exogenous implementation of NOx species increases the tolerance level against abiotic stresses in plants by improving the antioxidant defense system. Other researchers have reported that NOx positively contributes to the seed germination and plant growth^44,45^ and that exogenous NOx induces the germination of seeds for several abiotic stresses or decreases the seed dormancy^38^. The plasma generated species O_3_, OH radicals, and H_2_O_2_ cause oxidative stresses in plants, and NOx activates defense responses. cPTIO, a commercially available NO scavenger, is commonly and effectively used to elucidate the responses of NOx on plant growth and nutritional content^37^. In this study, the effect of NOx on the growth, yield and nutritional quality of soybean sprout, was assessed by a passive method using cPTIO. To ensure the presence of any unreacted cPTIO in the irrigation water a stoichiometric amount (150 µM) of this reagent was added. Hence, no free cPTIO remained present in the irrigation water which could affect on the growth and the nutritional quality of bean sprout produced in this study either in positive or negative manner. Therefore, it can be stated that cPTIO neither mediate nor shows any toxicity on soybean sprout growth and its nutritional quality. Addition of NO scavenger shows that the germination percentage remains unaffected (Fig. 3) that only slows down the rate of germination regardless of the type of irrigation water. Cultivation with plasma-discharged water showed a higher reduction in the size and total weight of soybean sprouts when cPTIO was added (Fig. 1SA and B and Table 1). Kitazaki *et al*. (2010) reported that seed growth stimulation occurred with plasma treatment due to the generation of ROS^46^. Sarinont *et al*. (2016) demonstrated that OH and O radicals could play an important role in growth enhancement^28^. The growth enhancement by OH and O radicals might be induced by NOx production, which shows a protective effect against mild oxidative stress^47^. However, during hydroponic beansprout production, the presence of cPTIO in plasma-discharged water showed an adverse impact on sprout growing because of the interruption of endogenous NOx generation. Fig. 2S shows the weight changes in the three different sections of the sprout for different types of irrigation water used during cultivation. Cotyledon and radicle weight did not show any remarkable variations for the different types of irrigation water used (*p* > 0.05). The measured hypocotyl weight was remarkably increased (*p* < 0.05) when the irrigation was performed with plasma-discharged water instead of tap water. The addition of cPTIO decreased the hypocotyl and radicle weights, and a more pronounced decrease in hypocotyl weight was observed with the presence of cPTIO in plasma-discharged water compared to in tap water. The decrease in radicle weight in presence of cPTIO was not significant for the different types of irrigation water used in this study (*p* > 0.05). However, in presence of cPTIO the overall reduction in weight and length occurred, which indicated the presence of NOx in the irrigation water improves the growth rate and yields of soybean sprout for same duration of farming.

A soybean sprout has three different parts: the cotyledon, the hypocotyl, and the radicle. Differences in these structures were considered during the analysis of nutrient content because the nutrient content varies by part^48^. Among the nutrients, the ascorbate, asparagine, and GABA contents of soybean sprouts are of great interest due to their health benefits and radical scavenging ability. To evaluate the role of irrigation water on the nutritional amount of soybean sprouts, measurements were performed after 4 days of cultivation with two types of irrigation water in the water recycling system. Figure 5(A–C) shows the content of ascorbate, asparagine, and GABA in soybean sprouts in three different parts. Figure 5A shows that the addition of cPTIO reduced the ascorbate content in the cotyledons, indicating that NOx in plasma-discharged water may show a vital role in the accumulation of ascorbate in the cotyledons. On the other hand addition of cPTIO increases the ascorbate content in the hypocotyl and radicle which indicated that ascorbate accumulation in the hypocotyls and radicle could be increased by oxidative stress or other unknown reasons that have not been studied yet. Some researchers have shown that asparagine plays a role in nitrogen mobilization during germination^49^ and accumulates in plant tissues subjected to environmental stress^50^. Other groups have shown that the asparagine content in plants increased with the addition of urea or ammonia as a nitrogen source in the cultivating water^49,50^. The presence of NOx increased the asparagine content in the cotyledons and hypocotyls of soybean sprouts. In contrast, the addition of cPTIO reduced the amount of asparagine in the cotyledons and hypocotyls by scavenging the NOx from the cultivation water. In the case of radicle, the type of cultivating water did not show any major variation in the amount of asparagine. The addition of cPTIO significantly reduced the asparagine content, representing that NOx radicals play a vital role in increasing asparagine content in soybean sprouts. However, a decrease in GABA content in the cotyledons in the presence of cPTIO indicated that the presence of NOx in the plasma-discharged water stimulated the accumulation of GABA in the cotyledons. The nutrient accumulation phenomenon is very different on the different parts of the soybean sprout. Further studies are required to understand these differences in nutrient accumulation in the various parts of soybean sprouts.

H_2_O_2_ generated in plants under various abiotic stresses showed a positive response in terms of plant growth and the exogenous addition of H_2_O_2_ caused physiological changes and nutrient accumulation. In other studies, a concentration of 0.5 mM H_2_O_2_ promoted the accumulation of bioactive molecules in radish sprouts^51^, and 10 mM stimulated germination for maturing wheat seed^52^. H_2_O_2_ at a concentration of 4.6 µM contained in plasma-discharged water was lower than the amount of exogenous H_2_O_2_ used in previous studies required to induce physiological changes in sprout cultivation treated with H_2_O_2_ alone. However, the effect of H_2_O_2_ on plant growth may vary depending on several factors, such as H_2_O_2_ concentration, type of seed, irrigation atmosphere, etc. There is a possibility of the presence of dissolved O_3_ gas when the plasma device remains activated. Hence O_3_ may play some role on the bean sprout cultivation; O_3_ is an abiotic stress factor that causes oxidative stress at the cellular level. Higher doses of O_3_ have resulted in leaf injury and loss of plant productivity^53^. Modest dosages of O_3_ may increase plant resistance by stimulating decontamination, antioxidants and additional process for protection. Rozpądek *et al*. (2013) used 70 ppb of O_3_ for fumigation to enhance the growth of *Brassica oleraces* seedlings^54^.

This study is a novel application of plasma-discharged water recycling technology for the nutrition rich, hygienic and economic hydroponic production of soybean sprouts. Oxides of nitrogen present in plasma-discharged water played an important role on overall growth enhancement of soybean sprout as well as hypocotyl elongation and asparagine accumulation. ROS with the influence of NOx in plasma-discharged water may have participated in ascorbate and GABA accumulation in the hypocotyls and radicle of the soybean sprout. In this water recycling system for hydroponic production, soybean sprouts were watered every 30 m throughout the production process. With this frequent watering schedule, no additional washing/cleaning step was required after harvesting; hence, the water consumption was reduced to a greater extent, making this technology comparatively economical. Routine DBD plasma treatment of the irrigation water for every 12 hours kept the irrigation water free from any microbial growth. Hence, we can conclude that plasma-discharged water recycling technology can be implemented in the soybean sprout production industry as an economical technology to produce nutritionally rich and hygienically grown soybean sprouts in a shorter period of time. However, advance studies are necessary to evaluate the optimum concentration of plasma-discharged species, in irrigation water required for cultivation and to understand the mechanism of nutrition accumulation in soybean sprouts.

## Materials and Methods

### Plasma system for cultivating water

**Figure 6:**
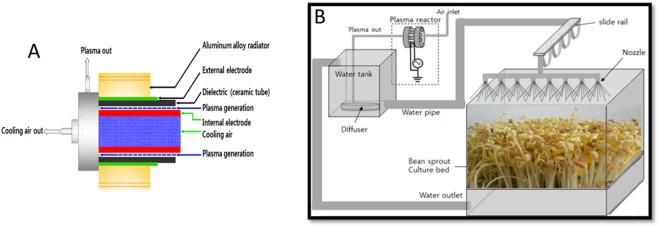
(**A**) Schematic diagram of the DBD electrode for plasma generation. (**B**) Plasma-discharged water recycling system used in this study.

To generate plasma a dielectric barrier discharge (DBD) electrode was used. As a dielectric material a ceramic tube (30 mm internal diameter) was used and was placed between the external and internal electrodes. The external electrode was cylindrical (40 mm outer diameter, 36 mm inner diameter, stainless steel (SUS304)) and contained a cylindrical internal electrode (26 mm outer diameter, 22 mm inner diameter, SUS304) under the dielectric with a gap (2 mm) (Fig. 6A). The air flow was adjusted to 5 l/min thru a controller towards the plasma generating area. The generated plasma jet fled by a diffuser (58 cm^2^ area, flat and round shape, Angelaqua, Korea) inside the water and produces bubble. This plasma-discharged water (PDW) was used for cultivation. The complete plasma discharged water recycling technology is shown in Fig. 6B.

### Plant material and growing conditions

Soybean seeds (*Glycine max* L. Merrill) were purchased from a farm located in Jeju, Korea in 2015 and stored at 4 °C until used. The soybean seeds were waterlogged in tap water for 30 m after that drained and washed with tap water. Four washed seeds were placed in sets of 100 seed cultivators (Chungsiru SC-9000, Shinchang INC, Osan, Korea) and watered for 30 seconds every 30 minutes with a nozzle throughout cultivation. The recycled irrigation water was treated for 5 minutes in every 12 hours during cultivation. The schematic diagram of plasma-discharged water recycling system is shows in Fig. 6B. Soybean growth was carried out at 20 °C in the dark and harvested after 4 days.

### Determination of radical’s concentrations

The concentration of dissolve O_3_ was found by a UV O_3_ display (Model 620, Ebara Jitsugyo, Japan). The O_3_ containing water was circulated via a drain present in the bottom of the device. The OH radicals in the water were determined with an OH radical scavenger, known as terephthalic acid^20^. The H_2_O_2_ in the water was determined by H_2_O_2_ assay kit (Amplex^®^, Molecular Probes, Eugene, OR, USA). To measure the amount of NOx generates in the plasma discharged water nitric oxide assay kit (QuantiChrom, BioAssay Systems, Hayward, CA, USA) was used.

### NO scavenger

In order to scavenge the dissolved nitric oxide in water produced by plasma device, 2-(4-Carboxyphenyl)-4,4,5,5-tetramethylimidazoline-1-oxyl-3-oxide potassium salt (c-PTIO, Sigma-Aldrich, MO, USA) was used. c-PTIO and NO reacts 1:1 and hence the stoichiometric amount of c-PTIO was used in this study^55^. The concentration of cPTIO used in this study was 150 µM in order to obtain NOx free environment.

### Microbiological analysis

After 24 hours of water recycling, the irrigation water was collected, and the microbial growth and reduction potential of the DBD plasma were evaluated. The inactivation effect of *Salmonella* Typhimurium ATCC14028 inoculated in irrigation water was also evaluated. One cluster of *S*. Typhimurium (KCCM, Seoul, Korea) cultured on plate count agar (PCA, BD, Sparks, MD, USA) at 37 °C for 24 hours, was inoculated in TSB broth (BD) at 37 °C for 15 hours. A centrifugation at 10,000 × *g* for 10 m was performed for harvesting and washing the culture. The bacteria pellet was suspended again about 6–7 log CFU/ml in 1 L irrigation water for 1 day. An aliquot (1 ml) of the water was pipette out and directly moved to 10 µl of 10% (w/v) sterile Na_2_S_2_O_3_ solution as a neutralizer during regular intervals of plasma treatment. The total quantity of aerobic bacteria was determined using in plate count agar PCA (BD), and xylose-lysine-desoxycholate XLD (BD) was used for *Salmonella* enumeration. The cultures were incubated aerobically at 37 °C for 24–48 hours. The microbial counts are expressed as log CFU g^−1^.

### Yields of soybean sprouts

After 4 days of cultivation, the yield parameters (i.e., germination percentage, weight, and the length of hypocotyl and radicle) were measured. The germination percentage was obtained by counting the amount of germinating seeds compared to the entire amount of seeds after the imbibition of 24, 48 and 72 hours. The fresh weight of each sprout was measured after removing the cotyledon chaff and moisture, and the outcomes were denoted as g/100 g of sprout. The individual weight in each part, including the cotyledon, hypocotyl, and radicle, was measured. The hypocotyl and radicle lengths were measured with a ruler according to the procedure described in (Rui *et al*.)^9^.

### HPLC analysis Ascorbate, asparagine and GABA content

For the HPLC analysis, each part of a fresh soybean sprout was homogenized with a 10% methanol/phosphate solution and was filtered through a syringe-driven filter with a 0.45 µm hole size before inoculation. An aliquot (20 µl) of the analyte passed through HPLC (Thermo Dionex ultimate 3000) fortified with a UV-Vis sensor (264 nm) and a column (C-18 column, inno C18 column 4.6 × 250, 5 µm, Innopia, Korea). The composition of mobile phase was purified water with 0.05 M (NH_4_)H_2_PO_4_ (A) and methanol (B). Ascorbate was detected via comparing the relative retention times and UV (264 nm) spectrum of a standard compound, and ascorbate was distinguished by an external standard technique. Each part of a soybean sprout was extracted with 75% ethanol for 24 hours after sonication for 1 hour. The extract was cleaned by a 0.2 µm membrane filter. To identify amino acid a procedure followed that described in Henderson *et al*.^56^.

### Statistical analysis

All the data obtained in this study were examined by SAS program (SAS Institute Inc.). Every measurement had 3 replicates. If ANOVA displayed important treatment properties, Duncan’s multiple range trial was done for comparing the averages at *p* < 0.05.

## Electronic supplementary material

Supplementary Figures
