# Supplementary material for: Roles of oxides of nitrogen on quality enhancement of soybean sprout during hydroponic production using plasma discharged water recycling technology
Source: Sci Rep. 2018 Nov 15;8:16872. doi: 10.1038/s41598-018-35385-5 (PMC6237935; doi:10.1038/s41598-018-35385-5)

Roles of oxides of nitrogen on quality enhancement of soybean sprout during hydroponic production using plasma discharged water recycling technology

Eun-Jung Lee^1^, Muhammad Saiful Islam Khan^2^, Jaewon Shim^2^, Yun-Ji Kim^2,3,*^

^1^ Faculty of Food Science and Biotechnology, College of Life Science, Sejong University, Seoul 05006, Republic of Korea

^2^ Division of Food Safety and Distribution, Korea Food Research Institute, Wanju-Gun, Jeollabuk-Do, 55365, Republic of Korea

^3^ Department of Food Biotechnology, University of Science and Technology, Daejeon, 305-350, Republic of Korea

**Figure 1S.** Photograph of the produced soybean sprouts after 2 days (A) and 4 days (B) for two different types of irrigation water. Control represents the irrigation with tap water.

A


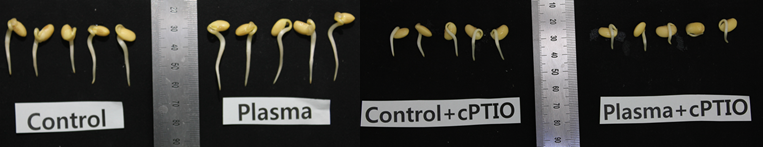


B


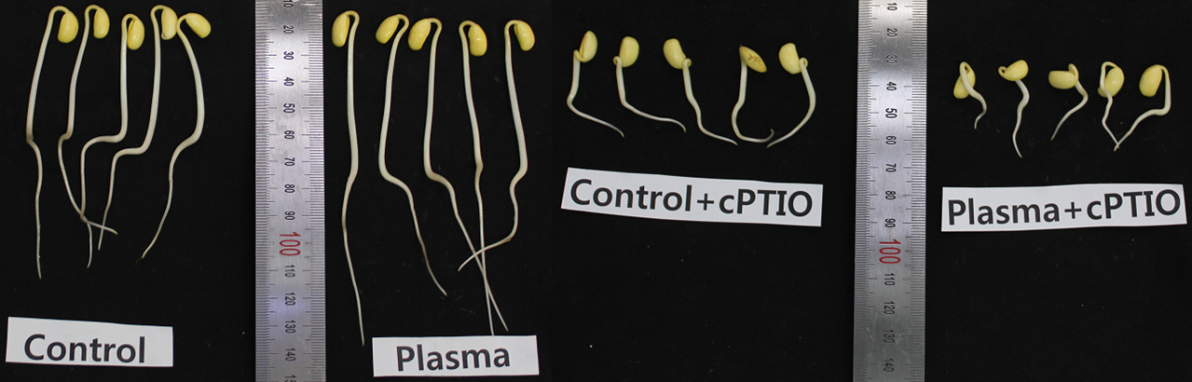


**Figure 2S.** The changes in the individual weights of each plant part after 4 days depending on the type of irrigation water. Treatments with different letters for the same plant part of the soybean sprout were significantly different based on Duncan’s multiple range test (*P<*0.05).


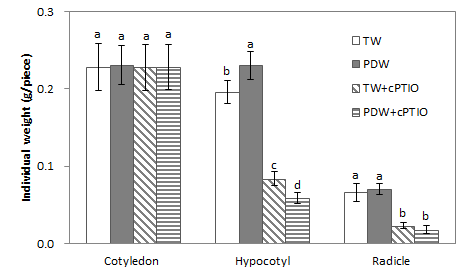

Supplement: Supplementary file 1 — Supplementary Figures [file 41598_2018_35385_MOESM1_ESM.docx]
